# Supplementary material for: Identification of PARP-7 substrates reveals a role for MARylation in microtubule control in ovarian cancer cells
Source: eLife. 2021 Jan 21;10:e60481. doi: 10.7554/eLife.60481 (PMC7884071; doi:10.7554/eLife.60481)
Supplement: Supplementary file 2. — The synthesis of many of these NAD+ analogs was reported in Gibson et al., 2016. Most of the compounds can be purchased from the BIOLOG Life Science Institute (https://www.biolog.de). a The number assigned to the NAD+ analog for this study. b Abbreviation assigned by BIOLOG Life Science Institute and used herein. For the clickable analogs: alkyne = contains an alkyne group, or azide = contains an azide group for use in copper-catalyzed alkyne-azide cycloaddition (‘click’) reactions. c BIOLOG Life Science Institute catalog number. [file elife-60481-supp2.docx]

**Table S2. Clickable NAD^+^ analogs used in this study.**

| **NAD^+^ Analog Number *^a^*** | **Chemical Name** | **Abbreviation *^b^*** | **BIOLOG Catalog Number *^c^*** | **CAS Registry Number** |
| --- | --- | --- | --- | --- |
| 1 | β-Nicotinamide-8-butylthio-N⁶-propargyl adenine dinucleotide | 8-BuT-6-Parg-NAD^+^ *(alkyne)* | N/A | N/A |
| 2 | β-Nicotinamide-8-(3-butynylthio) adenine dinucleotide | 8-Bu(3-yne)T-NAD⁺  *(alkyne)* | N 055 | 2022926-15-2 |
| 3 | β-Nicotinamide-8-(2-azidobenzylthio) adenine dinucleotide | 8-oN₃-BT-NAD⁺ *(azide)* | N 057 | Pending |
| 4 | β-Nicotinamide-8-(4-azidophenacylthio) adenine dinucleotide | 8-pN_3_-PAcT-NAD^+^  *(azide)* | N 053 | Pending |

The synthesis of many of these NAD^+^ analogs was reported in Gibson *et al.* (2016). Most of the compounds can be purchased from the BIOLOG Life Science Institute (<https://www.biolog.de>).

***^a^*** The number assigned to the NAD^+^ analog for this study.

***^b^*** Abbreviation assigned by BIOLOG Life Science Institute and used herein. For the clickable analogs: *alkyne* = contains an alkyne group, or azide = contains an azide group for use in copper-catalyzed alkyne-azide cycloaddition (“click”) reactions.

***^c^*** BIOLOG Life Science Institute catalog number.
