# Supplementary material for: Identification of PARP-7 substrates reveals a role for MARylation in microtubule control in ovarian cancer cells
Source: eLife. 2021 Jan 21;10:e60481. doi: 10.7554/eLife.60481 (PMC7884071; doi:10.7554/eLife.60481)
Supplement: Transparent reporting form [file elife-60481-transrepform.pdf]

eLIFE
1st Floor
24 Hills Road
Cambridge CB2 1JP, UK
P 01223 855340
W elifesciences.org
T @elife

**_eLife's_ transparent reporting form**

We encourage authors to provide detailed information *within their submission* to facilitate the interpretation and replication of experiments. Authors can upload supporting documentation to indicate the use of appropriate reporting guidelines for health-related research (see EQUATOR Network), life science research (see the BioSharing Information Resource), or the ARRIVE guidelines for reporting work involving animal research. Where applicable, authors should refer to any relevant reporting standards documents in this form.

If you have any questions, please consult our Journal Policies and/or contact us: editorial@elifesciences.org.

**Sample-size estimation**

- You should state whether an appropriate sample size was computed when the study was being designed
- You should state the statistical method of sample size computation and any required assumptions
- If no explicit power analysis was used, you should describe how you decided what sample (replicate) size (number) to use

Please outline where this information can be found within the submission (e.g., sections or figure legends), or explain why this information doesn't apply to your submission:

We did not use power analyses to determine the replicate numbers. The rationale for deciding on the number of replicates can be found in the next section ("Replicates"). The information on replicates can be found in the Methods and Figure Legends, as indicated in the next section.

**Replicates**

- You should report how often each experiment was performed
- You should include a definition of biological versus technical replication
- The data obtained should be provided and sufficient information should be provided to indicate the number of independent biological and/or technical replicates
- If you encountered any outliers, you should describe how these were handled
- Criteria for exclusion/inclusion of data should be clearly stated
- High-throughput sequence data should be uploaded before submission, with a private link for reviewers provided (these are available from both GEO and ArrayExpress)

Please outline where this information can be found within the submission (e.g., sections or figure legends), or explain why this information doesn't apply to your submission:

eLife Sciences Publications, Ltd is a limited liability non-profit non-stock corporation incorporated in the State of Delaware, USA, with company number 5030732, and is registered in the UK with company number FC030576 and branch number BR015634 at the address 1st Floor, 24 Hills Road, Cambridge CB2 1JP | August 2014

For analyses of public data sets, we used all of the samples available in the data set. The number of samples is indicated in the Methods and the Figure Legends.

For cell-based endpoint assays (e.g., expression, growth, migration, invasion, IP-Western, IF), we used n=3 biological replicates defined as temporally independent platings of cells from different stocks. We chose this number since it is customary in the literature for these types of assays. Some assays (e.g., RT-qPCR) also contained technical replicates within each biological replicate (direct, side-by-side repeat with the same sample). The number of samples is indicated in the Methods and/or the Figure Legends.

For genomic assays (e.g., RNA-seq), we used n=2 biological replicates defined as temporally independent platings of cells from different stocks. We chose this number based on ENCODE guidelines. Depth of sequencing was based on ENCODE guidelines. The number of samples is indicated in the Methods and the Figure Legends. The raw data can be accessed by the reviewers as follows:
To review GEO accession GSE153395:
Go to https://www.ncbi.nlm.nih.gov/geo/query/acc.cgi?acc=GSE153395
Enter token gbclgouifxgtxcz into the box

For the mass spectrometry proteomic assays (e.g., RNA-seq), we used n=2 biological replicates defined as temporally independent platings of cells from different stocks. The number of samples is indicated in the Figure Legend. The raw mass spectrometry data are provided in Supplemental Tables S2 and S3.

**Statistical reporting**

- Statistical analysis methods should be described and justified
- Raw data should be presented in figures whenever informative to do so (typically when N per group is less than 10)
- For each experiment, you should identify the statistical tests used, exact values of N, definitions of center, methods of multiple test correction, and dispersion and precision measures (e.g., mean, median, SD, SEM, confidence intervals; and, for the major substantive results, a measure of effect size (e.g., Pearson's r, Cohen's d)
- Report exact p-values wherever possible alongside the summary statistics and 95% confidence intervals. These should be reported for all key questions and not only when the p-value is less than 0.05.

Please outline where this information can be found within the submission (e.g., sections or figure legends), or explain why this information doesn't apply to your submission:

For analyses of public data sets, the statistical tests are indicated in the Figure Legends.

For cell-based endpoint assays (e.g., expression, growth, migration, invasion, IP-Western, IF) the statistical tests are indicated in the Figure Legends.

For the Gene Ontology (GO) analyses, the statistical tests are shown in the Figures.

For the mass spectrometry proteomic assays, he number of samples is indicated in the Figure Legend. the statistical tests are shown in the are shown in Supplemental Tables S2 and S3.

eLife Sciences Publications, Ltd is a limited liability non-profit non-stock corporation incorporated in the State of Delaware, USA, with company number 5030732, and is registered in the UK with company number FC030576 and branch number BR015634 at the address 1st Floor, 24 Hills Road, Cambridge CB2 1JP | August 2014

(For large datasets, or papers with a very large number of statistical tests, you may upload a single table file with tests, Ns, etc., with reference to sections in the manuscript.)

**Group allocation**

- Indicate how samples were allocated into experimental groups (in the case of clinical studies, please specify allocation to treatment method); if randomization was used, please also state if restricted randomization was applied
- Indicate if masking was used during group allocation, data collection and/or data analysis

Please outline where this information can be found within the submission (e.g., sections or figure legends), or explain why this information doesn't apply to your submission:

Not applicable.

**Additional data files ("source data")**

- We encourage you to upload relevant additional data files, such as numerical data that are represented as a graph in a figure, or as a summary table
- Where provided, these should be in the most useful format, and they can be uploaded as "Source data" files linked to a main figure or table
- Include model definition files including the full list of parameters used
- Include code used for data analysis (e.g., R, MatLab)
- Avoid stating that data files are "available upon request"

Please indicate the figures or tables for which source data files have been provided:

**Figure 2 – RNA-seq:**
Supplemental Table S1
To review GEO accession GSE153395:
Go to: https://www.ncbi.nlm.nih.gov/geo/query/acc.cgi?acc=GSE153395
Enter token gbclgouifxgtxcz into the box

**Figure 6 – mass spectrometry:**
Supplemental Tables S3 and S4
To review MassIVE accession number MSV000086611:
Go to: https://massive.ucsd.edu/ProteoSAFe/static/massive.jsp
• To view the dataset's web page (including title, description, and metadata), log in at the upper right corner of the page.
Username for web access: MSV000086611_reviewer
password if asked: KrausLab_LPMass
• To view the dataset's files, log in to the MassIVE FTP server with this URL:
ftp://MSV000086611@massive.ucsd.edu
Username for FTP access: MSV000086611
